# Epidemiological Study of Rickettsial Infections in Patients with Hypertransaminemia in Madrid (Spain)

**DOI:** 10.3390/ijerph6102526

**Published:** 2009-09-28

**Authors:** Lourdes Lledó, Rosario González, María Isabel Gegúndez, María Beltrán, José Vicente Saz

**Affiliations:** 1 Department of Microbiology and Parasitology, Faculty of Medicine, University of Alcalá, Ctra. Madrid-Barcelona Km 33.6, Alcalá de Henares 28871, Spain; E-Mails: rgonzalezpa@yahoo.es (R.G.); isabel.gegundez@uah.es (M.I.G.); mbeldub@yahoo.es (M.B.); josev.saz@uah.es (J.V.S.); 2 Department of Microbiology, Hospital “Príncipe de Asturias”, Ctra. Madrid-Barcelona Km 33.6, Alcalá de Henares 28871, Spain

**Keywords:** Rickettsiae, hypertransaminemia, liver dysfunction, prevalence, Spain

## Abstract

A retrospective analysis was performed to detect anti-rickettsial antibodies in the serum of patients with hypertransaminemia of unknown etiology, and in that of healthy members of the general population of Madrid (Spain). Among 143 patients 16 (11.2%) were positive for anti-*R. conorii* IgG antibodies and 7% for *R. typhi*. PCR analysis was performed in patients with IgM antibodies. Among 143 healthy subjects from the general population, seven (4.9%) were positive for anti-*R. conorii* IgG antibodies, and 2.8% for *R. typhi*. These results show that anti-rickettsial antibodies are more commonly detected in patients with hypertransaminemia than in healthy people.

## 1. Introduction

In recent years, the prevalence of infections with some of the species of rickettsia known to cause human disease has been increasing, and new rickettsial species causing human illness have been found [1]. Further, long-known rickettsia of undetermined pathogenicity have now been associated with disease. Several factors may be responsible for the emerging status of these infections, including climate and ecological changes [2] causing animal reservoirs to move from their original habitats and come into closer contact with pets - and therefore with humans. Changes in human demographics and the boom in outdoor activities in developed countries may also have led to greater exposure of humans to arthropod vectors.

The diseases caused by rickettsia vary from mild to severe in their presentation, with fatality ranging from 0 to over 30%. Infection with any member of the spotted fever (SFG) or typhus group (TG) usually involves fever and exanthema and is commonly associated with some liver dysfunction which in rare cases can be severe [3,4]. The symptoms of the emerging rickettsioses appear not to be so typical, which renders them difficult to diagnose (a problem that may well lead to underdiagnosis) [5], but even these infections are commonly associated with liver dysfunction.

The aim of the present work was to improve our knowledge of rickettsial infections in Spain. Over the last few years the country has seen an increase in their incidence; indeed, new rickettsial pathogens such as *R. felis* [6] have been discovered, and new diseases as such as DEBONEL/TIBOLA have been recognized [7]. In this work, the seroprevalence of anti-rickettsial antibodies in patients with liver dysfunction, identified by high transaminase levels, was measured and compared to that seen in healthy subjects.

## 2. Patients and Methods

**Serum specimens:** Serum was collected from 143 patients (patient group; 101 males, 42 females; age range 1–103 years, mean 43.8 years, standard deviation [SD] 18.3 years) with hypertransaminemia (serum aspartate aminotransferase [AST] > 38 U/L and/or alanine aminotransferase [ALT] > 35 U/L; see Table 1).

Patients with known hepatic viral infections, who were alcoholic, or who were undergoing treatment with hepatotoxic drugs, were excluded. The selected patients were under clinical study for different reasons: 99 for known hypertransaminemia of indeterminate origin without other clinical manifestations, three for acute hepatitis, six for fever, 13 for chronic liver disease, two for thrombocytopenia, and three for fatigue and weakness. The rest had come for a routine check-up of their health.

**Figure 1.**
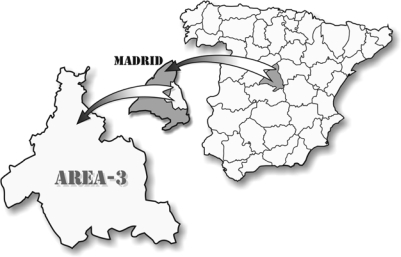
The geographical area of the study.

Serum was also collected from 143 members of the general population (control group; 101 males, 42 females; age range 1–99 years, mean 41.3 years, SD 18.88 years) with normal ALT and AST values. These subjects came from the same geographical region as the patients (Figure 1) (i.e., Health Area 3 of the Madrid Region, which is home to the Príncipe de Asturias University Hospital).

The medical history and occupation of the patients and controls, their area of residence, travel history, and their history of contact with animals and arthropod bites were all recorded. All serum samples were maintained at −20 °C until analysis. All subjects gave their informed consent to be included in the study in compliance with the ethical standards of the Human Experimentation Committee of the University of Alcalá de Henares and the Helsinki Declaration of 1964 (as revised in 2004).

**Serological tests:** Sample sera were analyzed using the indirect immunofluorescence assay (IFA) described by Phillip *et al.* [8]. *Rickettsia conorii* (strain Malish 7) and *R. typhi* (strain Wilmington) were used as antigens. These were propagated in Vero E6 cells (ATCC CRL 1586) and fixed on spot slides. The fluorescein-labeled conjugates used were rabbit anti-human IgG and rabbit anti-human IgM serum (Sigma, St Louis, MO), diluted 1/128 in PBS containing Evan’s blue. Briefly, two-fold dilutions of each serum sample were added to the antigen spots and incubated in a humidity chamber for 30 min at 37 °C. After washing, the conjugates were added to each. The slides were then incubated for 30 min, washed, and examined using a BH2 Olympus fluorescence microscope (10 × 40). Positive and negative control sera were also examined; spots of uninfected Vero E6 cells were used to provide negative control antigens. Sera showing a typical pattern of fluorescence at titers of ≥1:128 for IgG and of ≥1:64 for IgM were deemed positive.

## 3. Molecular Methods

DNA was extracted from the serum of IgM-positive patients using the COBAS AmpliPrep^®^ system (Roche Diagnostics, GmbH, Mannheim, Germany) according to the manufacturer’s recommendations. Nested PCR assays targeting the rickettsial genes for citrate synthase (*gltA*) [9] using the primers CS415/CS1220 and RpCs1258/Rp877 that amplify a 381-bp fragment, and 190-kDa protein (*ompA*) gene [9,10] using Rr190.70p /Rr 190.602n and FW1/RV2 primers which amplifying a 492-bp fragment. To prevent DNA contamination and the carry-over of amplified products, sterile tools were used at all times in each step of the analysis (DNA extraction, preparation of the reaction mixture, and amplification and analysis of the PCR products); each step was performed in separate work areas. A negative control (Milli-Q water) was included all amplifications.

**Statistical analysis:** The differences in proportions in 2-way tables were analyzed using the χ^2^ or Fisher exact test. Significance was set at <0.05.

## 4. Results

Sixteen patients with hypertransaminemia (total 11.2%; females 4.8%, males 14%) had IgG antibodies for *R. conorii*. The age of the seropositive subjects ranged from 25 to 81 years (mean 50 years, SD 13.3 years). Ten patients with hypertransaminemia (total 7%; females 9.5%, males 5.9%) had IgG antibodies for *R. typhi*. The age of these seropositive subjects ranged from 1 to 68 years (mean 35 years, SD 17.6 years). Two patients were shown to have antibodies to both rickettsial species. No difference was noted in prevalence of infection in the rural and urban areas (13% vs. 9% for *R. conorii*, and 9.2% vs. 4.5% for *R. typhi*). Among the patients, no significant differences were found in seroprevalence with respect to sex or age.

Table 2 shows the clinical blood test results for all the seropositive patients (13 patients came to the clinic for known hypertransaminemia, one for fever, one for lymphocytosis, one for abdominal exanthema, one for abdominal pain, and the remainder for a routine check-up), and the patient media values and range of transaminase levels were 110.2 U/l and 90-2150 U/l for ALT and 107.7 U/l and 97-1480 U/l for AST respectively.

Four of the seropositive patients were immigrants. One of the patients referred to contact with cats; another was homeless. The remaining subjects had no history of contact with animals or arthropod bites, and had no occupational risks.

Only two patients were positive for IgM antibodies (also they had IgG antibodies): one for *R. conorii* antibodies (that of a 59 year-old man with abdominal pain, lymphopenia, hyperbilirubinemia, and elevated phosphatase alkaline [FA], lactate dehydrogenase [LDH] and gamma-glutamyl transferase [GGT]); the other for *R. typhi* antibodies (that of a 27 year-old man with elevated GGT). The molecular assay, however, did not detect rickettsial DNA.

In the control group, IgG antibodies to *R. conorii* were found in seven serum samples (total 4.9%; females 7.1%, males 4%). The mean age of these subjects was 55 years (SD 15 years; range 32–81 years). IgG antibodies to *R. typhi* were found in four serum samples (total 2.8%; females 4.7% and males 2%). The mean age of these subjects was 39 years (SD 16 years; range 19–86 years). One sample contained antibodies to both rickettsial species. Again, no difference was seen in prevalence of infection between the rural and urban areas (6.6% vs. 3% for *R. conorii* infection, and 4% vs. 1.5% for *R. typhi* infection) and respect to sex or age. The seropositive subjects had no history of contact with animals or arthropod bites, and had no occupational risks.

The prevalence of seropositivity for *R. conorii* in the patient and control groups was significantly different (p < 0.05; χ^2^ = 3.84), with more positive results among the patients. Table 3 shows the titers of IgG antibodies for the seropositive subjects.

## 4. Discussion

The rickettsia have a universal distribution, but our knowledge on the epidemiology of these organisms and their health impacts differs according to geographical region. These bacteria are associated with emerging diseases, and those associated with new rickettsial species are currently being investigated [11]. The spectrum of illness caused by rickettsia is very wide and the symptoms of infection in different areas may vary from those of classic SFG and TG infections. However, hepatic manifestations have been well described not only in SFG [3,12–16] and TG [4,5,17] infections, but also in clinical cases caused by emergent strains [7,18]. Liver damage is manifested as transaminase elevation and biological signs of cholostasis. Hepatic granulomas are also characteristic of infection [19] and rickettsiosis should be considered in the differential diagnosis of hepatitis [20]. When a rickettsial infection is suspected, liver function tests should always be performed. Some infections, however, may remain hepatically silent [15].

Emerging diseases caused by new strains of rickettsia have been recorded in Spain, including DEBONEL/TIBOLA caused by *R. slovaca* [7] and a typhus like fever caused by *R. felis* [21]. Further, the number of clinical SFG and TG infections has increased over the last few years in this country [22–24]. Studies in the general population, mainly for SFG, have tried to determine the circulating strains involved using molecular techniques, and to isolate them in animal reservoirs and vectors. Studies have also been undertaken to characterize the clinical manifestations of infection by emergent species.

The aim of the present study was to determine the prevalence of rickettsial infection among patients with liver dysfunction, and to determine whether there were differences that seen in the general population. Only two antigens (one SFG and one TG) were used since the different species of rickettsia show high levels of cross reactivity [25] (reducing the chance of missing any positive result). The results confirm the presence of SFG and TG infection in the study zone. The rate for both TG- and SFG-type infection in the general population was lower than that reported recently from other areas from Spain, e.g., prevalences of antibodies to *R. conorii* of 8% [26] and to *R. typhi* of 3.7% [27] have been reported from the northeast, and of 8.7% for *R. conorii* [28] and 3.8% for *R. typhi* [28] from the south. Similar figures are reported from Greece [29] (prevalence for *R. conorii* 7.9% and for *R. typhi* 2%, and most North African [30] countries (5–8% for *R. conorii* and 0.5–4% for *R. typhi*).

The present results showed no differences in prevalence according to sex, age, or origin (rural vs. urban). To date, *R. conorii* infection has more commonly been reported in rural areas, and *R. typhi* in urban areas. However, in recent years, studies in Spain [27] and in other places have reported this situation to be changing. This may be due to alterations in human demographics and behavior (movement to semi-rural areas, more outdoor activities) leading to more contact between wild animals and humans and their pets.

The results show that in the study area the patients with hypertransaminemia more commonly possessed antibodies to rickettsia than the members of the control group. The patients showed a higher prevalence of both SFG and TG infection, and their antibody titers were higher. Anti-rickettsial IgM antibodies were also detected in two patients, although they had no recent history of illness compatible with classical rickettsial infection and no rickettsial DNA was detected in their serum. One year later, seventeen patients saw their transaminase levels normalize and two continued with high levels of ALT; five patients did not keep further appointments and were lost to analysis.

This is the first time that the prevalence of rickettsial infection among patients with impaired liver function has been examined, and the data obtained seem to indicate that the present group could have greater contact with these organisms. It may therefore be appropriate to screen for rickettsia in such patients, even though they do not present all the signs and symptoms characteristic of rickettsiosisal, because the list of newly discovered rickettsiae has grown rapidly in recent years and the clinical manifestations of these infections may be atypicals. However, it is important to mention that there are cross-reaction with other conditions, for example autoimmune hepatitis, therefore other serological studies with more specific assays are planned to further confirm the prevalence of rickettsial-antibodies among patients with liver dysfunction with the aim to avoid false positives.

In summary, rickettsioses are emerging diseases in Spain. Large-scale epidemiological surveillance is therefore needed, and clinical data should be diligently collected in order to help in the diagnosis of infection.

## Figures and Tables

**Table 1.** Patient ALT and AST levels.

	**Patients n (%)**	**Mean**	**Range**

ALT >35 U/L	112 (78.3)	97.4	36–2150
AST >38 U/L	57 (39.8)	91.3	39–1480

49 patients (34.2%) had high ALT and AST levels.

**Table 2.** Analytical abnormalities in seropositive patients.

**Clinical findings**	***R. conorii* N (%)**	***R. typhi* N (%)**

Lymphocytosis	4 (25%)	5 (50%)
Lymphopenia	3 (18.7%)	2 (20%)
Thrombocytopenia	2 (12.5%)	-
Hyperbilirubinemia	4 (25%)	2 (20%)
Elevated alkaline phosphatase	4 (25%)	4 (40%)
Elevated GGT	4 (25%)	2 (20%)
Elevated lactate dehydrogenase	3 (18.7%)	2 (20%)

**Table 3.** Titers for antibodies (IgG) to *R. conorii* and *R. typhi*.

**Groups**	**Titer 1/128**	**Titer 1/256**	**Titer 1/512**	**Titer 1/1024**	**Titer 1/2048**

Patient with Ab*-R. conorii*	6 (4.2%)	5 (3.5%)	5 (3.5%)	-	-
Patient with Ab*-R. typhi*	3 (2.1%)	4 (2.8%)	2 (1.2%)	-	1 (0.7%)

Control population with Ab-*R. conorii*	3 (2.1%)	-	4 (2.8%)	-	-
Control population with Ab-*R. typhi*	2 (1.2%)	1 (0.7%)	1 (0.7%)	-	-
